# Point Density Variations in Airborne Lidar Point Clouds

**DOI:** 10.3390/s23031593

**Published:** 2023-02-01

**Authors:** Vaclav Petras, Anna Petrasova, James B. McCarter, Helena Mitasova, Ross K. Meentemeyer

**Affiliations:** 1 Center for Geospatial Analytics, North Carolina State University, 2800 Faucette Dr., Campus Box 7106, Raleigh, NC 27695, USA; 2 Department of Forestry and Environmental Resources, North Carolina State University, 2820 Faucette Dr., Campus Box 8001, Raleigh, NC 27695, USA; 3 Department of Marine, Earth, and Atmospheric Sciences, North Carolina State University, 2800 Faucette Drive, Campus Box 8208, Raleigh, NC 27695, USA

**Keywords:** airborne lidar, laser scanning, point density pattern, nonuniform point distribution, geospatial mapping, remote sensing, surface topography

## Abstract

In spite of increasing point density and accuracy, airborne lidar point clouds often exhibit point density variations. Some of these density variations indicate issues with point clouds, potentially leading to errors in derived products. To highlight these issues, we provide an overview of point density variations and show examples in six airborne lidar point cloud datasets that we used in our topographic and geospatial modeling research. Using the published literature, we identified sources of point density variations and issues indicated or caused by these variations. Lastly, we discuss the reduction in point density variations using decimations, homogenizations, and their applicability.

## 1. Introduction

Point clouds acquired by airborne lidar have transformed how Earth’s ground surface, vegetation structure, and urban environments are mapped and analyzed. This has led to major advances in terrain modeling, flood prediction, coastal monitoring, forestry and ecosystem studies, and many other disciplines [1,2,3,4]. As with all data collection methods, the process of acquiring point cloud data introduces inconsistencies and errors. Although recent technological advancements aim to minimize the measurement errors, various issues are still present in current datasets. Additionally, datasets captured by legacy systems continue to be a valuable source for many studies detecting long-term changes of the environment [5,6,7,8]. It is, therefore, important to understand errors and anomalies in data acquired by both new and older data acquisition systems to ensure the proper processing, harmonization, and interpretation of point cloud datasets.

Although errors in lidar point clouds were extensively studied [9,10,11,12,13,14] and local variations of point density were recognized in the literature [15,16,17,18,19,20,21], there is less emphasis on studying these density variations and their potential impact on derived products [22,23,24,25,26,27]. Understanding point density variations is important, as lidar point cloud density measurements are used in numerous applications, such as estimating biomass [28], forest fuel mapping [29,30,31], subcanopy solar radiation modeling [32], and mapping forest structures [3,33,34,35,36]. Furthermore, products derived from point clouds such as 3D meshes and digital elevation models (DEMs) are influenced by the presence and absence of points [37]. Lastly, density anomalies may be an indicator of errors in point elevations and issues with other properties of the point cloud, such as missing or vertically unaligned swaths.

In this work, we provide an overview of point density variations and associated errors in airborne lidar point clouds, and discuss their impact on the derived products. We demonstrate the variations using six point cloud datasets (Table 1) used in our topographic and geospatial modeling research [5,33,38,39,40,41]: North Carolina (NC) Floodplain Mapping, 2015 [42], Wake County, 2013 [43], Nantahala 2009 by the National Center for Airborne Laser Mapping (NCALM) [44], and three coastal mapping datasets by NOAA, NASA, and USGS from 1999, 2003, and 2008 [45,46,47]. We reference the current literature to support the examination of the datasets, and to identify potential sources of density variations. Lastly, we discuss decimation and homogenization approaches for reducing point density variations and their appropriate use.

## 2. Point Density Variations

**Figure 1:**
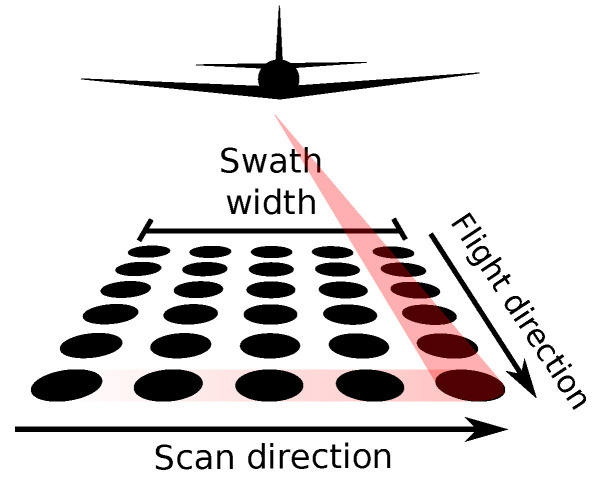
Conceptual illustration of airborne lidar scanning. Scan direction (scan line) is typically perpendicular to the flight direction. The area covered during scanning while an aircraft flies in a constant direction is called the swath or flight strip.

In an idealized case [48,49,50], an aircraft flies at a constant speed, direction, and altitude above ground level with zero roll, pitch, and yaw, and a known position. A line scanner aboard the aircraft scans the mapped area in the direction perpendicular to the line of flight. The scanner instantly captures a complete scan line with constant spacing between the points on the ground. The speed of the aircraft is constant to ensure that the point spacing in the scan line is the same as the spacing between the scan lines (Figure 1). Lastly, the aircraft flies in straight parallel lines separated by the width of the scan line (swath or flight strip), so that the individual swaths have minimal overlap, and cover the surveyed area without gaps. For a flat terrain without vegetation or other objects, the result would be a regular grid of points. However, none of these idealized procedures is fulfilled in real world conditions. Scanner type, flight plan execution, environmental conditions, data georeferencing, and the presence of vegetation and ground structures all influence airborne lidar data acquisition and the resulting variations in point density. The following paragraphs describe common density variations and related errors for airborne lidar point clouds.

### 2.1. Scan Patterns

**Figure 2:**
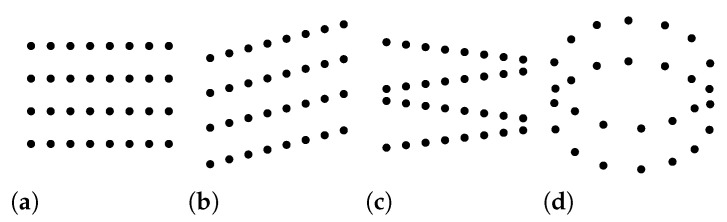
Scan patterns for basic types of sensors [49,52,53]: (**a**) line scanner or fiber scanner pattern; (**b**) rotating polygon or rotating mirror pattern; (**c**) oscillating or swinging mirror pattern; and (**d**) conical, elliptical, or Palmer scan pattern.

**Figure 3:**
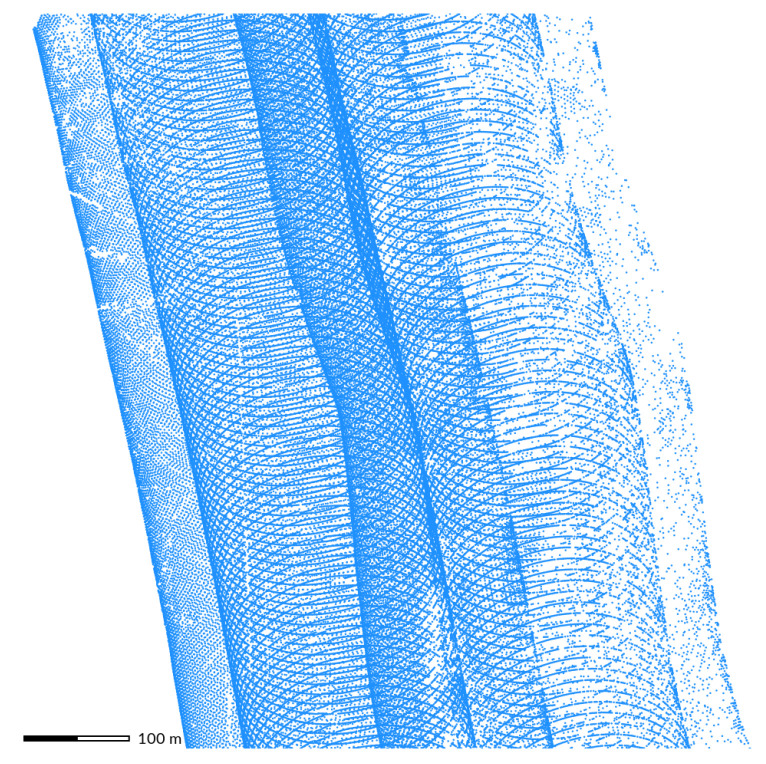
Point cloud from several overlapping swaths with an elliptical scan pattern (two swaths in the middle of the stripe of points). Elliptical scanning causes a higher point density at the edge of each swath. Dataset: NOAA/NASA/USGS 1999 [45].

The spatial pattern of points is largely dependent on the type of scanner (Table 2, Figure 2). Rotating mirror scanners and oscillating mirror scanners produce parallel scan lines (assuming constant flight direction) or a zigzag line [51]. While parallel scan lines result in equal point density (Figure 2a,b), a zigzag line always results in variable point density (Figure 2c). Fiber scanners capture the whole scan line at once, producing parallel scan lines that are perpendicular to the flight direction, thus potentially leading to more uniform point distribution [49,52]. Scanners with conical (elliptical) scan patterns (Figure 2d), such as Palmer scanners and some single-photon scanners, measure with a constant scan angle in all directions [53,54,55]. Consequently, each location is scanned twice from different angles, and the conical pattern causes very high density on the edges of each swath unless the scanner accounts for that by changing the speed of scanning. The 1999 dataset by NOAA/NASA/USGS [45] had several overlapping swaths, but clearly shows a conical (elliptical) pattern and the resulting high point density on the swath edges (Figure 3).

### 2.2. Variations along Scan Line

**Figure 4:**
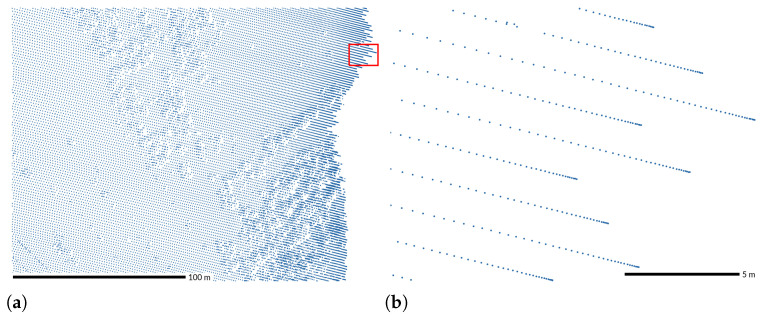
For some scanners, point density (**a**) increases towards the end of the scan line and (**b**) peaks at the very end. The extent of (**b**) is highlighted in (**a**). Only points with specific source ID, scan direction, and user data field are displayed to highlight the scan line. Dataset: NC Floodplain Mapping Program 2015 [42].

Point spacing in the direction of a scan line is influenced by the inner working of the scanner and is another source of scanning-related point density variations (Table 2). Older line scanners usually scan in equal angle steps, which results in higher point density in the middle of the scan line, and lower density toward the ends of the scan line [49]. On the other hand, newer scanners can achieve equal point spacing in the middle of the scan line, but anomalies can still occur at the end of a scan line due to the acceleration of the inner mechanical parts of mirror scanners. For example, the NC Floodplain Mapping Program dataset exhibited higher point density at the end of the scan line (Figure 4).

### 2.3. Banding

**Figure 5:**
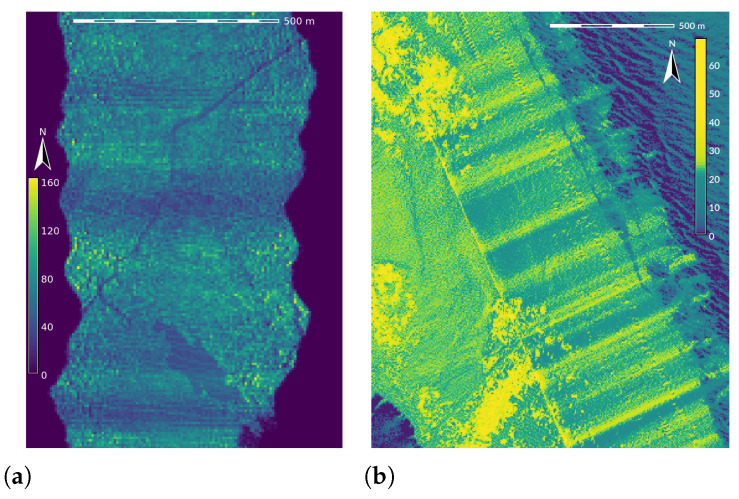
Variable aircraft speed and pitch results in areas with higher and lower point density along the flight line (here approximately north-south direction). Point density is expressed as the number of points per grid cell at 6 m resolution (**a)** and 5 m resolution (**b**). Darker areas indicate lower point density while lighter areas indicate high density. Datasets: NCALM 2009 [44] (**a**) and NOAA 2008 [47] (**b**).

Lidar scanners are usually synchronized with the speed of the aircraft to achieve constant density in all directions [56]. However, variable aircraft speed due to wind, when not accounted for by the scanner, results in stripes of higher and lower densities along the flight line [56]. These periodical changes in densities are called *banding* [37] and can be observed easily by computing the number of points per grid cell. Such an examination of the NCALM and NOAA datasets shows strong banding effect (Figure 5). To mediate speed-related banding, helicopters may be used to obtain high-resolution coverage with more uniform distribution of points because they can more easily control their speed [57].

Another cause of banding is a change in the pitch of the aircraft. In addition to irregular point density, this can also cause errors in measured elevation [37]. When the pitch value recorded by the navigation system is different from the actual pitch, the resulting point is assigned an incorrect position, causing artificially undulating terrain. To determine if the banding is just in density or also in elevation, the DEM can be compared to another DEM or a profile of the point density and the DEM can be examined.

### 2.4. Variations Due to Aircraft Altitude Variations

Point density is also influenced by the altitude of the aircraft above ground or sea level [58]. A lower altitude causes smaller point spacing in the scan line, resulting in variable point density when the aircraft altitude varies during the flight. Additionally, too low altitude may cause a swath to be too narrow to overlap with or touch an adjacent swath. For some scanners, such as those with an elliptical scan pattern, changes in altitude also influence spacing in the flight direction. For line scanners, the dynamically adjusted speed of scanning based on the altitude can mitigate density variations in all directions.

In mountainous areas, the changes of terrain elevation together with a constant altitude (height above sea level) of the aircraft cause variable densities in the higher and lower situated areas when the sensor is not adjusted. Similarly, steep slopes result in fewer points capturing the surface. Although the horizontal density stays the same in this case, there are fewer points covering a given surface in a sloped area in comparison to a flat area.

### 2.5. Swath Overlaps

**Figure 6:**
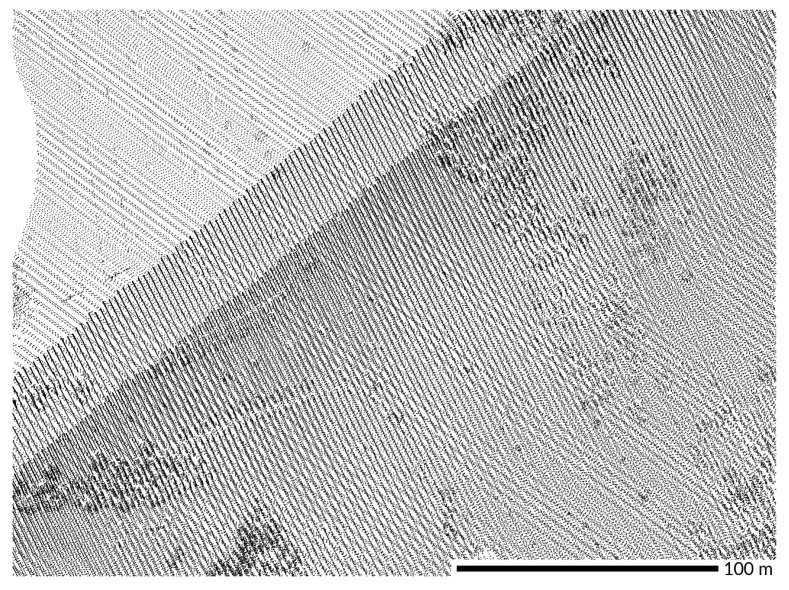
Example of a complex point distribution. The area is completely covered by one swath and partially covered by another. The border of the second one reveals the doubled scan pattern and higher density at the end of the scan line. There are bands of higher and lower densities in the direction of the flight (approximately diagonal direction). Other density variations are related to vegetation and the small area with missing points on the left is caused by a water body. Dataset: Wake County 2013 [43].

**Figure 7:**
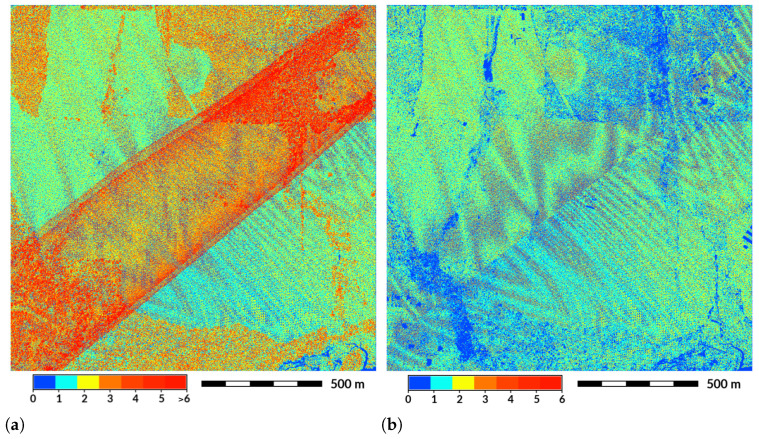
Swath overlap as the main source of variation in point density. (**a**) Overlap of two swaths in red and orange around the image diagonal. (**b**) An abrupt change in the pattern that follows a straight line (close to image diagonal) marks the transition between swaths and removed swath overlap in classified ground points. Additionally, both figures showed a Moiré effect in the point density. Figures show the number of points per grid cell at 1 m resolution. Dataset: Wake County 2013 [43].

Airborne lidar point clouds are collected in parallel swaths (passes). To ensure that there are no gaps in between swaths, individual swaths usually overlap, as in the Wake County [43] dataset (Figure 6 and Figure 7). Another reason for collecting overlapping swaths is to mitigate errors introduced by the acquisition of the position and orientation of the aircraft, which are used to derive point coordinates. Collecting swaths with overlap generates redundant information that can be used to align individual swaths by eliminating the horizontal and vertical time-dependent shifts [48]. These shifts, occurring due to systematic navigation system errors, can then be minimized via, for example, least-squares matching [56,59]. However, swath overlaps are a significant source of point density variations [60,61]. Therefore, overlaps are often removed from the dataset, especially if classification is performed, for example, classified ground points in the Wake County dataset do not include any swath overlaps (Figure 7). Although overlap removal eliminates the issue of higher point density, the new issue of having abrupt changes in the point density patterns arises.

**Figure 8:**
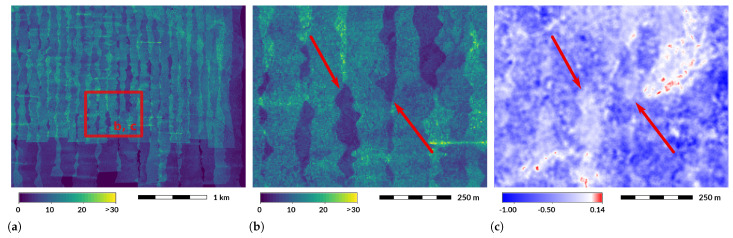
Places with low point density cause differences in skewness of point elevations. (**a**) Larger area showing two sets of north–south swaths. Color represents the number of points per square meter. (**b**) Dark violet areas represent places with low point density (<10 points per square meter). (**c**) Skewness of point elevations. Some areas with zero skewness (in white) are associated with areas with low point density. Most prominent places are marked by red arrows. Skewness is smoothed using circular 23 × 23 moving window to remove local extremes. Dataset: NCALM 2009 [44].

For vegetation-related metrics, such as the subcanopy solar radiation model [32], any variable point density at the swath overlap may be an issue. For example, the NCALM dataset had two sets of north–south swaths overlapping each other (Figure 8). As a result, the dataset contains areas with high point density caused by two or more swaths overlapping and areas with low point density caused by a gap in between the swaths in one of the sets. Derived skewness of point elevations, used in forestry applications [62], then shows both actual features (e.g., vegetation types) and artifacts representing the gaps (Figure 8).

**Figure 9:**
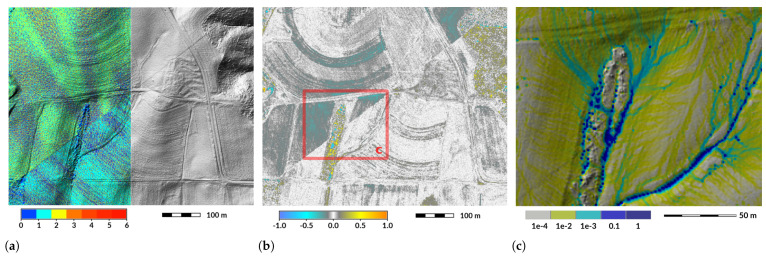
Error in swath elevation registration (a line close to the image diagonal): (**a**) Split view between point density and shaded relief (1 meter resolution, year 2013). (**b**) Elevation difference in meters; year 2015 minus year 2013. (**c**) Water discharge in $m^{3}\;s^{-1}$ simulated by SIMWE [65] with default parameters. Datasets: Wake County 2013 [43], NC Floodplain Mapping Program 2015 [42].

For terrain reconstruction, an abrupt change in density between two swaths may highlight an abrupt change in point elevations that are a result of uncorrected errors in positioning [63]. A positioning error that was not corrected by aligning swaths resulted in a flawed terrain reconstruction using the Wake County dataset. In the dataset, an abrupt change in elevation corresponds to an abrupt change in the point density pattern and is visible on the shaded relief model and when the dataset is compared with the NC Floodplain Mapping Program dataset (Figure 9). This is an issue for further time series analysis because it prevents direct comparison of time steps. The flawed terrain reconstruction causes issues for further analysis. For example, simulated water flow may follow the abrupt change in elevation instead of the actual terrain (Figure 9c).

Furthermore, the width of swath overlaps may vary. While modern scanning systems try to adjust the scan line and apply on-board checks, changes in the aircraft’s altitude and roll, and navigation errors may lead to overlap width variations or gaps (Table 2). Lastly, a difference in the number of swaths can cause point density differences between otherwise comparable datasets [64].

### 2.6. Moiré and Corduroy Effects

**Figure 10:**
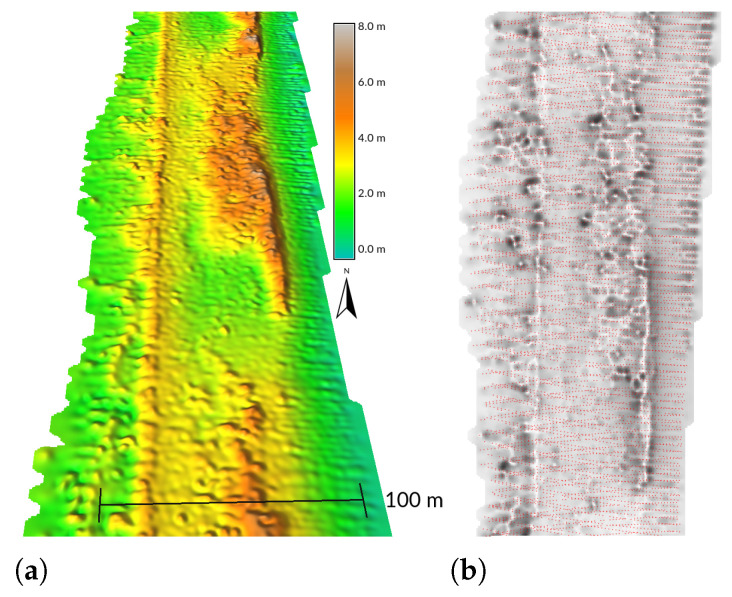
Corduroy effect visible on (**a**) the interpolated DEM visualized in 3D and (**b**) the source point cloud (red) overlaid with a DEM-derived skyview factor visualization [71] that shows the low places between corduroy ridges in dark gray. The DEM was interpolated at 0.5 meter resolution. Dataset: NASA/USGS 2003 [46].

When the number of points per grid cell is computed to spatially analyze the point density, the overlap of the grid pattern and the point pattern may cause a Moiré effect (pattern) in the resulting image of point density [66,67,68]. Wake County dataset shows the Moiré effect in the point density of classified ground points (Figure 7b). Additionally, when two alternating series of scan lines overlap as a result of swath overlap, scanning back and forth with a line scanner, or doubled scan pattern, a detailed digital elevation model contains stripes of higher and lower elevation when the points are not accurately georeferenced [66,69,70]. This Corduroy effect can be observed in the NASA/USGS dataset where two sets of scan lines cause corduroy lines in the east-west direction (Figure 10). While the corduroy effect can be grouped together with the Moiré effect into oscillatory patterns [50], unlike Moiré, the corduroy effect is associated with elevations of points rather than just their positions in relation to a 2D grid.

### 2.7. Surface Material Properties and Obstructions

**Figure 11:**
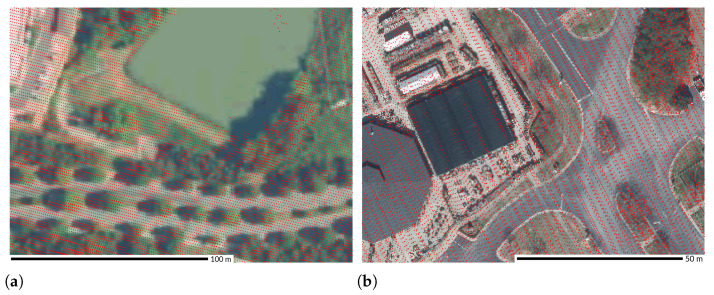
Areas without points caused by (**a**) a water body and (**b**) by a dark asphalt roof. Dataset: Wake County 2013 [43].

Other commonly recognized sources of lower densities or gaps in point clouds are signal noise, surfaces absorbing the pulses, and small obstructing objects such as small details on building roofs [72]. Depending on the wavelength used by the lidar sensor, some surfaces, mainly water and dark asphalt, return a weaker signal or do not return any pulses, which results in lower point return intensities or no recorded points at all. The surfaces that are most likely to suffer from these issues are water bodies, wetlands, and some types of roads and roofs. Examples from the Wake County dataset show almost no points for a water body and reduced number of points for a specific building roof (Figure 11).

### 2.8. Variations Due to Vegetation

**Figure 12:**
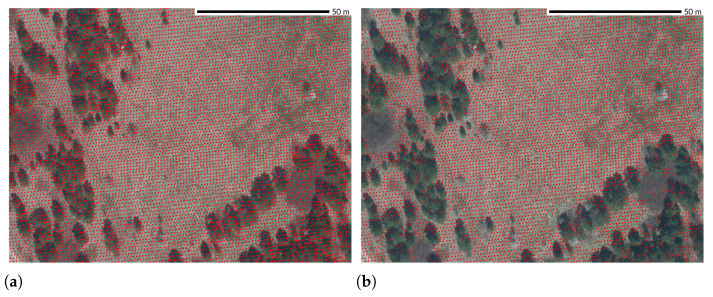
Horizontal point pattern in vegetated and nonvegetated areas: (**a**) Points from all returns had a regular pattern in open areas combined with irregular distributions in the vegetated areas. (**b**) Classified ground points in areas without vegetation had the original pattern, while in the areas with vegetation the distribution is much sparser. Dataset: NC Floodplain Mapping Program 2015 [42].

Density and spatial distribution of points in vegetated areas typically differ from nonvegetated areas due to occlusion or multiple returns in the canopy as opposed to a single return from the bare ground [53,62]. For example, the NC Floodplain Mapping Program dataset had a regular point pattern in nonvegetated areas; in vegetated areas, distribution is irregular, and classified ground points have lower density (Figure 12).

**Figure 13:**
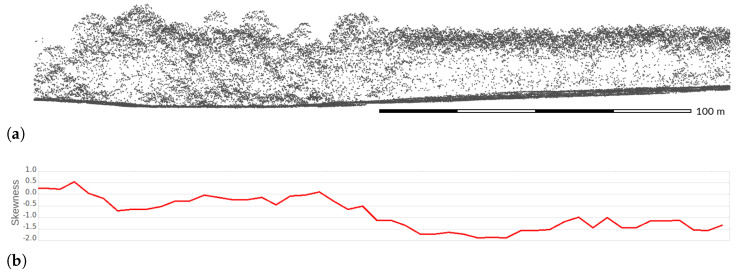
(**a**) Vertical distribution of lidar points in natural forest (**left**) and a planted forest (**right**) in a 220 m long and 30 m wide transect of a forested area. (**b**) Skewness of point elevations along the same transect without ground points. Dataset: NC Floodplain Mapping Program 2015 [42].

**Figure 14:**
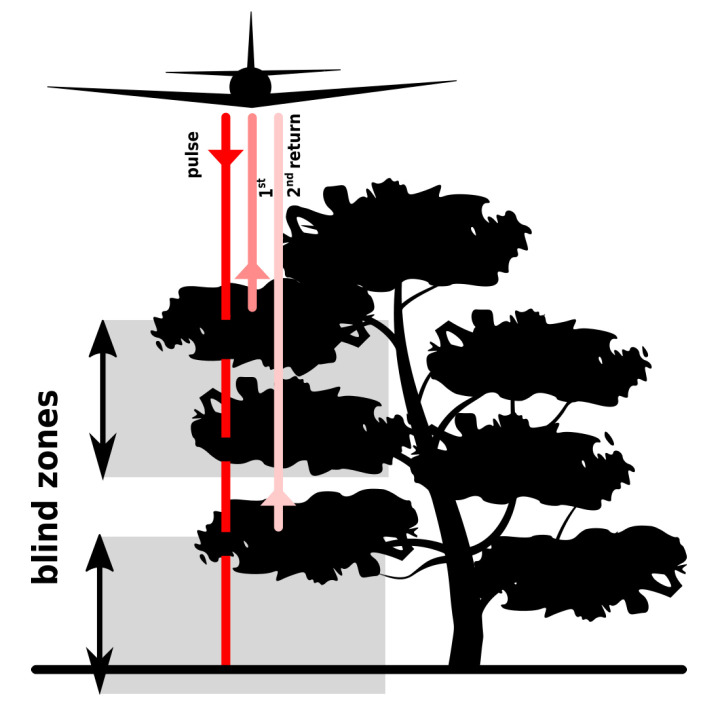
The blind-zone effect, i.e., no points recorded after a given record, may cause the omission of points in certain parts of the vegetation or on the ground. Emitted pulse penetrates the canopy and produces returns. The first return is from top of the canopy and is registered by a detector. Then, no further returns from the canopy are registered until the detector is ready to record another return (top gray rectangle). When the detector is ready to register again, the second return is recorded from the lower parts of the canopy. Again, no further returns from the canopy or ground are registered because the detector is not ready to record (bottom gray rectangle).

Point density is also variable in the vertical direction depending on several factors related to the structure of vegetation (which can be used for classification). For example, a planted forest is characterized by significantly skewed distributions in comparison to a natural one [73] that is visible on a transect from the NC Floodplain Mapping Program dataset (Figure 13). In the natural forest, lidar pulses penetrate the canopy and reveal complex structure and understory (Figure 13, left). In the planted forest, most of the points are concentrated in the canopy suggesting thick canopy and no understory, given the presence of the ground points (Figure 13, right). This difference can be used for classification to distinguish these two forest types. The forest type also influences the wintertime and summertime rates of penetration through the canopy [48]. Lastly, a so-called *blind zone* may cause gaps in vertical point distribution [63]. A blind zone is a distance where no subsequent returns are recorded after a previously recorded return due to a limited response time of the detector (Figure 14). For example, lidar sensor Leica ALS80-HP reports a blind zone distance of 2.8 m [74].

### 2.9. Ground Points

**Figure 15:**
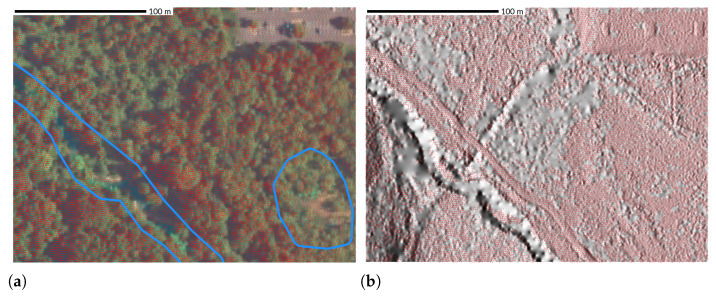
Lower density of ground points in vegetated areas or lack of points in wet areas usually causes these areas to be smoother than the rest of the area when points are interpolated into a DEM: (**a**) Lower density of all return points around a stream and in a vegetated, supposedly wet area (marked in blue). (**b**) Classified ground points (red) missing in some areas completely. The influence on the DEM becomes most apparent when the DEM is used to derive additional products such as the shaded relief here. Dataset: NC Floodplain Mapping Program 2015 [42].

The NC Floodplain Mapping Program dataset demonstrated that points classified as ground may have much higher variability in both pattern and overall density compared to the variability of all points in the point cloud. (Figure 12). Depending on the vegetation density, fewer pulses penetrate the vegetation all the way to the ground, resulting in lower ground point density in vegetated areas than that in nonvegetated areas [48,53]. The gaps may also be caused by buildings or objects that do not reflect the pulses. The interpolation of these gaps to derive a digital terrain model (DTM) representing ground surface results in smooth areas where the level of detail is generally lower than that in areas with full point coverage (Figure 15). Additionally, ground points classified by data providers often have points from overlapping swaths (scan lines) removed as in the case of Wake County dataset (Figure 7). Together with other ground point density variations, such as abrupt changes (Figure 9) or banding (Figure 5), occlusion-caused gaps (Figure 15) may indicate an issue with accuracy of resulting DTMs (Table 3).

### 2.10. Redundant Points

Point redundancy, i.e., whether certain points in a dataset are needed, depends on a particular application rather than being an inherent property of a point cloud. Point redundancy can be a result of several aforementioned local density variations such as scan line anomalies (Figure 4), banding (Figure 5), and swath overlaps (Figure 9). Additionally, modern sensors generate point cloud densities that exceed the needs of most applications resulting in evenly distributed redundant points, for example, a Geiger-mode sensor may produce 25 points per square meter [54]. Lastly, when combining multiple adjacent point clouds with inconsistent point densities into one spatially continuous dataset or multiple overlapping point clouds with inconsistent densities into a time series, points in higher density point clouds would be considered redundant.

For the creation of DTMs, the minimal point density depends on the desired resolution of the surface [75]. Even though high-precision DTMs generally do not need the full density of the current point cloud [24,40,76,77,78,79], redundant points do not negatively influence the resulting DTM. However, redundant points can significantly increase the processing time or storage requirements [80,81,82], causing practical issues for many applications.

## 3. Reducing Point Density Variations in Point Clouds

Point density variations in point cloud datasets, including point redundancy, can be mitigated or removed using various decimation and homogenization techniques. While decimation removes redundant points, homogenization makes point distribution more uniform. Both approaches are commonly used during point cloud processing to improve the point cloud properties for a particular application.

### 3.1. Applicability of Decimation and Homogenization

Decimation, also referred to as thinning, downsampling, or simplification, is a reduction in the number of points in a point cloud that preserves selected properties of the point cloud. While many decimations lead to homogenization of the point density across a point cloud, full or true homogenization may include adding points where density is low, i.e., filling. Decimations and homogenizations can be applied to one or more point clouds to harmonize point densities across a time series or spatially adjacent datasets.

Given the prevalence of point density variations, algorithms working with point clouds should consider the effect of these variations [72,83]. However, not all applications can or need to remove the density variations from the point cloud. For example, such metrics as above-ground biomass (AGB) estimates [28], leaf area density (LAD) distribution [35], subcanopy solar radiation model [32], and the analysis of point density percentiles used as a measure of crown density [36] would be negatively affected by adding or removing points.

Decimation is advantageous for deriving DTMs and other products from point clouds at different resolutions in order to reduce processing time and computing resources needed [81,84,85]. Additionally, decimation can remove density variations resulting from swath overlaps. On the other hand, deriving high resolution digital surface model (DSM) and reconstruction of 3D objects, such as buildings or full 3D models of trees [86,87], may benefit more from higher point density than the creation of (usually smooth) DTMs.

For point cloud classification, high point density is usually advantageous to distinguish ground surface points from points that represent returns from trees, buildings, and other objects [9,14,88], and to preserve sharp edges of the objects such as building roofs.

### 3.2. Decimation and Homogenization

**Figure 16:**
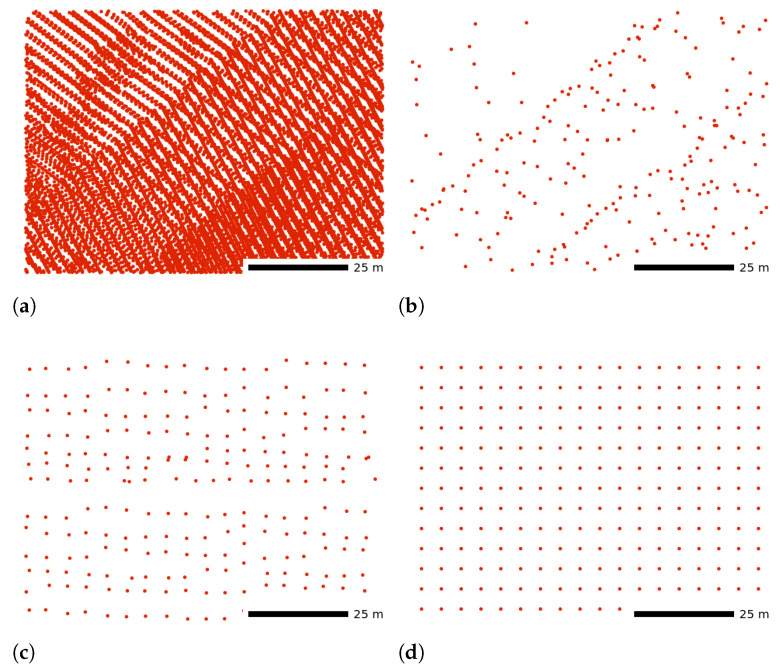
(**a**) Point cloud decimated using (**b**) count-based decimation keeping 1% of points (preserving every 100th point); (**c**) grid-based decimation preserving one point per 5 meter grid cell; (**d**) grid-based decimation with snapping to grid using the same 5 meter grid. Dataset: Wake County 2013 [43].

Decimation techniques range from simple ones, such as random sampling, to complex decimations based on shape of the objects described by the point cloud [82,89,90,91,92,93,94,95,96]. Random sampling can be based on the ordinal number of a point within the point cloud (count-based decimation). The resulting point cloud contains only every n-th point, but the overall point distribution remains the same; possibly preserving some of the variations such as higher density at the end of scan lines (Figure 16b). This technique works well for lidar point clouds when points are ordered by collection time and are used for creation of DTMs [97,98].

Grid-based decimation (Figure 16c) is based on binning of the points into a 2D or 3D raster (grid) and is similar to creating a raster image from a point cloud (as opposed to creating a subset of a point cloud). Decimation can also be based on dividing space using kd-trees, quad-trees, oct-trees, or Voronoi diagrams [99,100,101]. These decimations typically work within the neighborhoods of the points and take into account distances between the points, size of the neighborhood, or a number of points in the neighborhood. At the cost of higher complexity, algorithms focused on surfaces and curvatures better preserve objects represented by the point cloud [61,95,102].

Many decimation techniques, such as grid-based decimation, aim at the homogenization of the point density by, e.g., snapping to a grid (Figure 16d). However, decimations can make the distribution of points more regular only in the areas with redundant points. For full homogenization, resampling based on regular grids can add points to areas with low point density [100,101,103].

3D raster (grid) creation or space voxelization, i.e., replacing groups of points by 3D cubes or single points, is used by some 3D metrics and object reconstruction methods to remove redundant points while characterizing, e.g., forest canopy fuel properties [104], internal patterns of vegetation [33], fine-scale bird habitat [105], and detailed tree models [106,107]. These methods often use relative point counts based on absolute point counts in the neighborhood [3,40], thus reducing the overall data and potentially reducing the influence of variable point density in the point cloud.

## 4. Conclusions

Despite the advancements in airborne lidar technology, different types of point density variations and related errors are common in currently used datasets as the cost of equipment and repeated airborne surveys remains high. To bring attention to these issues, we provided an overview of the different types of density variations that may indicate errors in the acquired data and may lead to artifacts or distortions in the derived products. We demonstrated point density variations and related artifacts by examples from six point cloud datasets and by referenced literature. Further, we discussed how density variations can be reduced in a point cloud by various decimation and homogenization techniques, along with the limitations of those methods.

While some authors include point density analysis [23,25,108,109] or take into account local density variations when processing lidar point clouds [87,103,110,111,112,113,114,115], our overview further highlights the importance of point density analysis as a starting point for studies using lidar point clouds. Special care should be given to point clouds used as input for analytical methods that are highly influenced by point cloud density, such as many vegetation-related metrics.

## Figures and Tables

**Table 1:** Overview of the six airborne lidar datasets used for demonstrating density variations. All datasets are for parts of North Carolina (NC), USA.

Name	Year	Area	Collected by	Reference
NC Floodplain Mapping Program	2015	Selected counties in Piedmont	State of North Carolina	[42]
Wake County	2013	Wake County	Wake County government	[43]
NCALM	2009	Nantahala National Forest	NCALM	[44]
NOAA/NASA/USGS	1999	Coast of NC	NOAA, NASA, USGS	[45]
NASA/USGS	2003	Cape Hatteras	NASA, USGS	[46]
NOAA	2008	Coast of NC and Virginia	NOAA	[47]

**Table 2:** Point density variations related to scanner and flight.

Source of Density Variation	Possible Effect on Point Density
Scanner type	Change in overall pattern
Variable line scanner point spacing	Lower or higher density along the scan line
Variable speed or pitch	Stripes of higher and lower density in the flight direction (banding)
Variable altitude	Higher or lower overall density
Altitude change preventing swath overlap	Gaps without points
Roll change preventing swath overlap	Gaps without points
Missing or additional swath due to navigation error	Gaps without points or higher point density
Missing or additional swath due to error in flight planning	Gaps without points or higher point density

**Table 3:** Errors in interpolated DEM, their relations to density, and their causes.

Cause of DEM Error	Possible Indication in Density or Pattern	Effect in DEM
Occlusion, low reflectivity	Gaps, very low density	Smooth areas (missing information)
Pitch changes and recording errors	Dense stripes	Elevation banding (waves)
Vertically unaligned swaths	Abrupt changes in pattern	Abrupt changes in elevation (break lines)
Vertically unaligned scan lines	Moiré effect, double scan lines	Corduroy effect (stripes)

## Data Availability

The airborne lidar data we used include NC Quality Level 2 lidar data (NC QL2) for North Carolina, USA collected by North Carolina Floodplain Mapping Program in 2015 [42] and Wake County lidar data for Wake County, North Carolina, USA collected in 2013 [43]. Further, we use coastal lidar data NOAA/NASA/USGS, 1999 [45] collected by National Oceanic and Atmospheric Administration (NOAA), National Aeronautics and Space Administration (NASA), and U.S. Geological Survey (USGS) in September 1999 in a post-Hurricane Floyd survey. The coastal dataset NASA/USGS, 2003 [46] from the pre-hurricane Isabel survey in 2003 was collected with the NASA/USGS Experimental Advanced Airborne Research Lidar (EAARL) [116] and the NOAA, 2008 [47] dataset was collected in March 2008 using the NOAA March Integrated Ocean and Coastal Mapping (IOCM) equipment. The Nantahala 2009 dataset [44] was obtained from OpenTopography. Lidar data acquisition and processing was completed by the National Center for Airborne Laser Mapping (NCALM). NCALM funding was provided by NSF’s Division of Earth Sciences, Instrumentation and Facilities Program EAR-1043051. All datasets are available online except the Wake County dataset, which is available from the Wake County Geographic Information Services (GIS) department. GRASS GIS software [117,118] used to create visualizations and compute densities and decimation is available online under GNU General Public License.

## Abbreviations

The following abbreviations are used in this manuscript:

AGB	Above-ground biomass
DEM	Digital elevation model
DSM	Digital surface model
DTM	Digital terrain model
EAARL	Experimental Advanced Airborne Research Lidar
IOCM	Integrated Ocean and Coastal Mapping
LAD	Leaf area density
NASA	(U.S.) National Aeronautics and Space Administration
NCALM	(U.S.) National Center for Airborne Laser Mapping
NC	North Carolina, USA
NOAA	(U.S.) National Oceanic and Atmospheric Administration
NSF	(U.S.) National Science Foundation
QL2	Quality Level 2
SIMWE	Simulation of water and erosion
USGS	U.S. Geological Survey
USA (U.S.)	United States of America
